# Supplementary material for: Precise gene regulation through transcriptional repression is essential for Plasmodium berghei asexual blood stage development
Source: Nat Commun. 2026 Jan 3;17:1508. doi: 10.1038/s41467-025-68222-1 (PMC12891473; doi:10.1038/s41467-025-68222-1)
Supplement: Supplementary file 1 — Supplementary information [file 41467_2025_68222_MOESM1_ESM.pdf]

Supplementary Figures

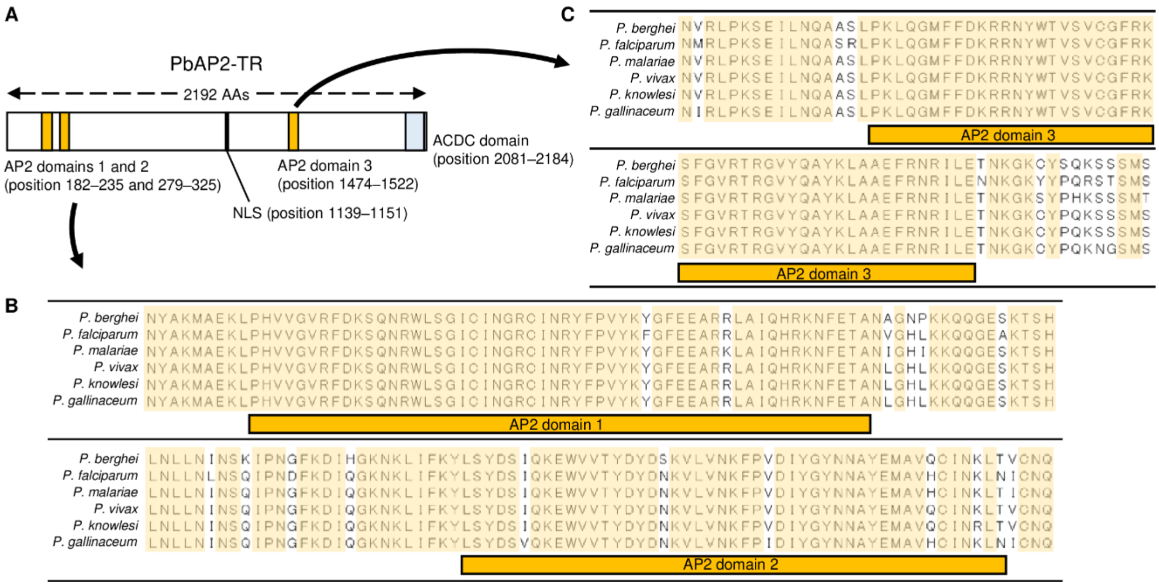

Fig. S1. Structural features of PbAP2-TR.

(A) Schematic illustration of PbAP2-TR. The regions of an AP2 domain are indicated by yellow. The nuclear localization signal (NLS) was predicted using cNLS Mapper (http://nls-mapper.iab.keio.ac.jp/cgi-bin/NLS_Mapper_form.cgi) and is indicated with a black bar. (B) Alignment of amino acid sequences for the two tandem AP2 domains of PbAP2-TR orthologs in *Plasmodium* using the ClustalW program in Mega X (*P. berghei*, PBANKA_0909600; *P. falciparum*, PF3D7_1139300; *P. malariae*, PmUG01_09048500; *P. vivax*, PVP01_0940100; *P. knowlesi*, PKNH_0937300; *P. gallinaceum*, PGAL8A_00366800). Amino acids conserved in all orthologs are highlighted by yellow. The regions of an AP2 domain are indicated by yellow boxes. (C) Alignment of amino acid sequences for the third AP2 domain of PbAP2-TR orthologs in *Plasmodium*.

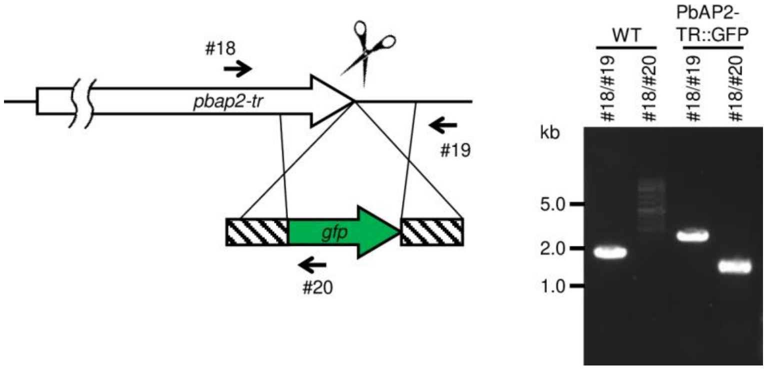

Fig. S2. Genotyping of PbAP2-TR::GFP.

Schematic illustration of gene editing at the *pbap2-tr* locus is shown on left. A gel image from the genotyping PCR analysis is shown on right. The primer numbers are listed in Table S8. Source data are provided as a Source Data file.

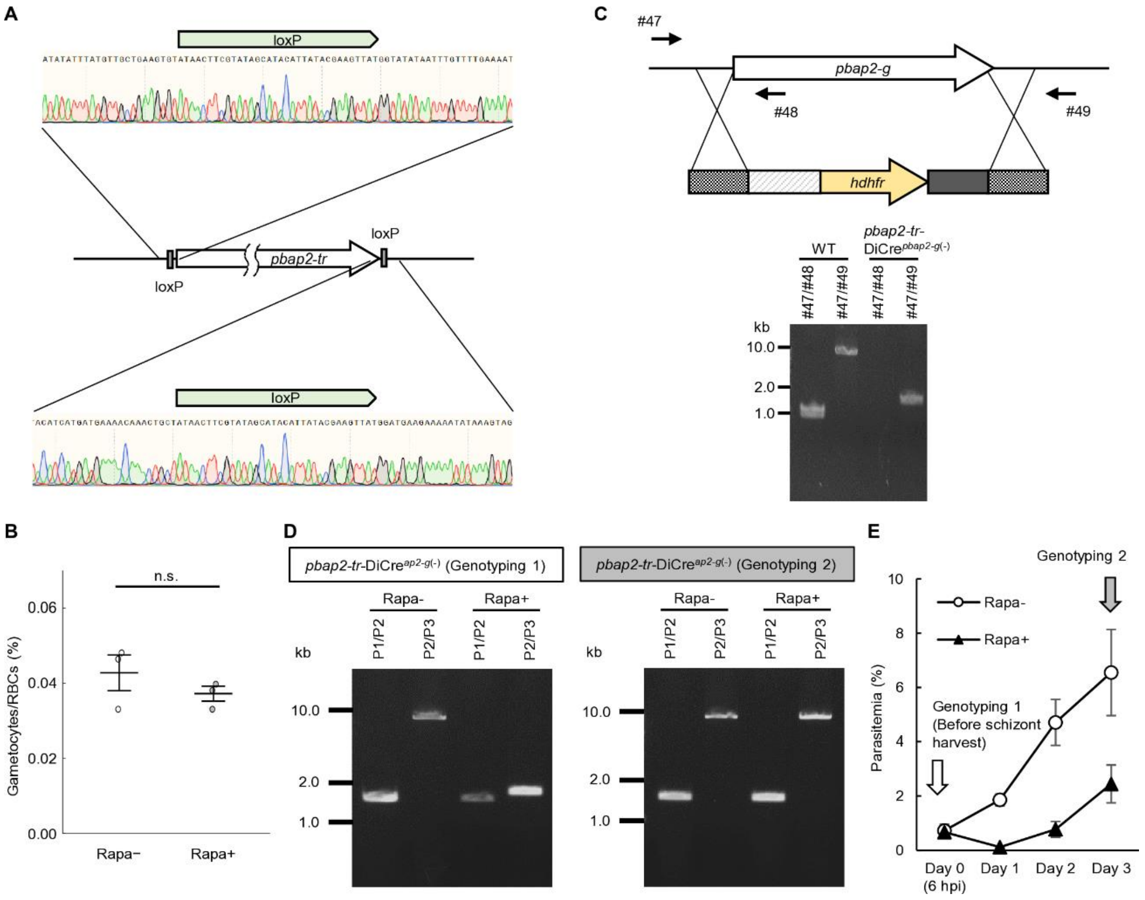

Fig. S3. Generation of of *pbap2-tr*-DiCre and *pbap2-tr*-DiCre$^{ap2\text{-}g(\text{-})}$.

(A) Genotyping of *pbap2-tr*-DiCre. Sangar sequence results confirming insertion of loxP at 5′- and 3′-side of *pbap2-tr* are shown on top and bottom. A schematic illustration of the *pbap2-tr* locus in *pbap2-tr*-DiCre is shown between them. (B) The number of gametocytes/red blood cells (RBCs) in *pbap2-tr*-DiCre$^{\text{Rapa}-}$ and *pbap2-tr*-DiCre$^{\text{Rapa}+}$ on day 1 (%). Lines indicate the mean values and SEM (n = 3). (n.s.: not significant, two-tailed Student's t-test, *p*-value > 0.05.) (C) Genotyping of *pbap2-tr*-DiCre$^{pbap2\text{-}g(\text{-})}$. Schematic illustration of gene editing at the *pbap2-g* locus is shown on top. A gel image from the genotyping PCR analysis is shown on bottom. The primer numbers are listed in Table S10. (D) Representative genotyping PCR analysis for *pbap2-tr*-DiCre$^{ap2\text{-}g(\text{-})_\text{Rapa}-}$ and *pbap2-tr*-DiCre$^{ap2\text{-}g(\text{-})_\text{Rapa}+}$ at 16 h after starting culture (Genotyping 1, left) and on day 3 after inoculating them into mice (Genotyping 2, right) from three biologically independent experiments. Primers used are illustrated in Fig 1B. (E) Parasite growth of *pbap2-tr*-DiCre$^{ap2\text{-}g(\text{-})_\text{Rapa}-}$ and *pbap2-tr*-DiCre$^{ap2\text{-}g(\text{-})_\text{Rapa}+}$ *in vivo*. Parasitemia was assessed by Giemsa staining. Error bars indicate SEM from three biologically independent experiments. Time points for Genotyping 1 and 2 are indicated by arrows. Source data are provided as a Source Data file.

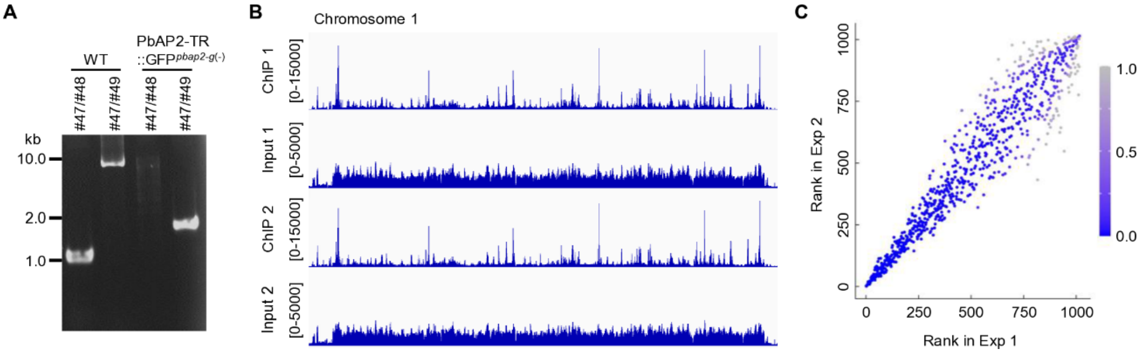

Fig. S4. ChIP-seq analysis using PbAP2-TR::GFP$^{pbap2\text{-}g(\text{-})}$.

(A) Genotyping of PbAP2-TR::GFP$^{pbap2\text{-}g(-)}$. PbAP2-TR::GFP$^{pbap2\text{-}g(-)}$ was derived from PbAP2-TR::GFP. *pbap2-g* was disrupted by the same strategy illustrated in Fig S2C. The primer numbers are listed in Table S10. (B) Integrative Genomics Viewer images for the PbAP2-TR ChIP-seq experiments 1 and 2 on chromosome 1. Both ChIP and input data are shown. Histograms show the raw read coverage of mapped sequence data normalized by library size (bin size = 10 bp). Scales are indicated in square brackets. (C) IDR1D analysis comparing the ChIP-seq experiments 1 and 2. The rank of peaks according to their *q*-value for each experiment is plotted against each other. The Irreproducible Discovery Rate (IDR) of each peak was represented using a gradient color scale. Source data are provided as a Source Data file.

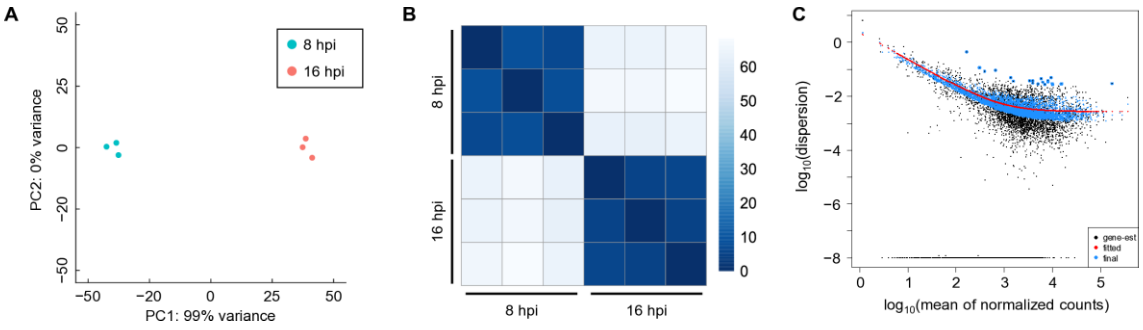

Fig. S5. Differential expression analysis between 8 hours post-injection (hpi) and 16 hpi using the *pbap2-g* knockout (KO) parasites.

(A) Principal component analysis of high-throughput RNA sequencing (RNA-seq) data using *pbap2-g* KO parasites at 8 and 16 hpi. RNA-seq analyses at each time-point were performed in biological triplicate. (B) Sample-to-sample distance heatmap of the 8 and 16 hpi RNA-seq data. (C) Dispersion plot of the 8 and 16 hpi RNA-seq data. Gene-wise estimates (gene-est), fitted estimates using the parametric fitting type (fitted), and maximum a posteriori dispersion estimates (final) are shown.

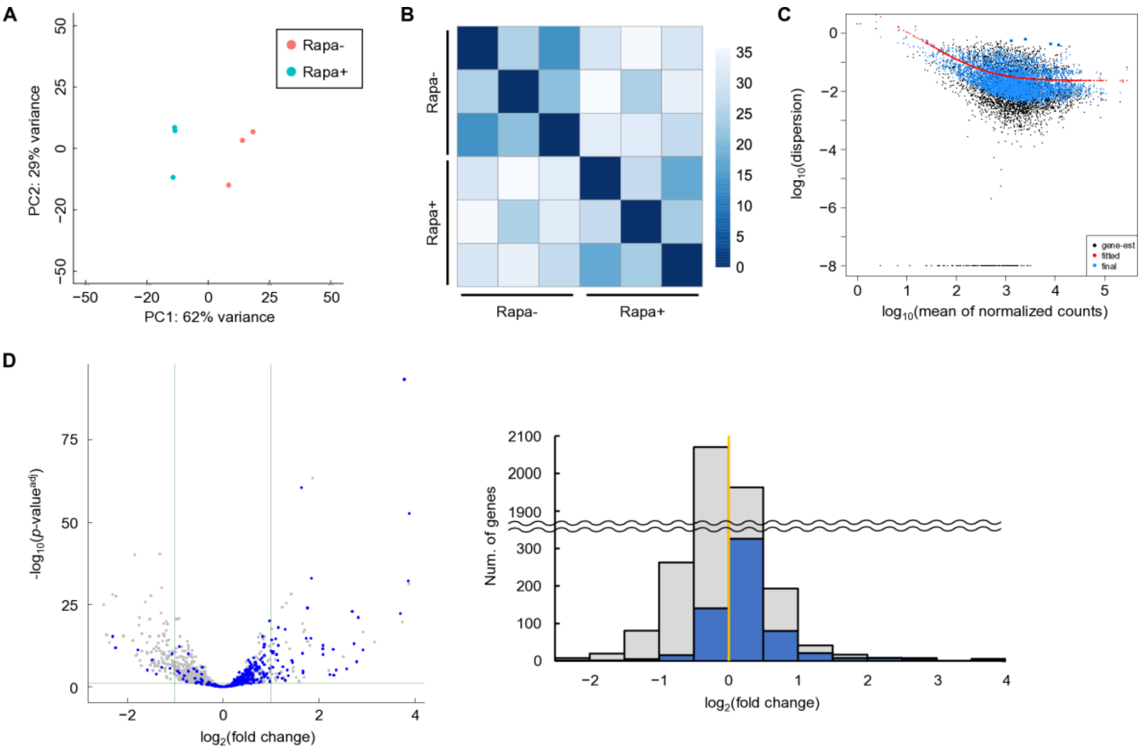

Fig. S6. Differential expression analysis between *pbap2-tr*-DiCre^{Rapa-} and *pbap2-tr*-DiCre^{Rapa+}.

(A) Principal component analysis of high-throughput RNA sequencing (RNA-seq) data from *pbap2-tr*-DiCre^{Rapa-} and *pbap2-tr*-DiCre^{Rapa+} at 12 hours post-injection (hpi). RNA-seq analyses of each sample were performed in biological triplicate. (B) Sample-to-sample distance heatmap of the *pbap2-tr*-DiCre^{Rapa-} and *pbap2-tr*-DiCre^{Rapa+} RNA-seq data. (C) Dispersion plot of the *pbap2-tr*-DiCre^{Rapa-} and *pbap2-tr*-DiCre^{Rapa+} RNA-seq data. Gene-wise estimates (gene-est), fitted estimates using the parametric fitting type (fitted), and maximum a posteriori dispersion estimates (final) are shown. (D) Volcano plot showing the differential expression of genes between *pbap2-tr*-DiCre^{Rapa-} and *pbap2-tr*-DiCre^{Rapa+} at 12 hpi (left). Blue dots represent the target genes of PbAP2-TR. *p*-values adjusted for multiple testing with the Benjamini-Hochberg procedure (*p*-value^{adj}) were calculated using DESeq2. The horizontal line indicates *p*-value^{adj} of 0.05 and the two vertical lines indicate the log$_2$(fold change) of 1 and −1. Histogram on right shows the number of genes against the log$_2$(fold change) values. The number of PbAP2-TR targets for each bin is indicated in blue. The yellow line indicates a log$_2$(fold change) of 0.

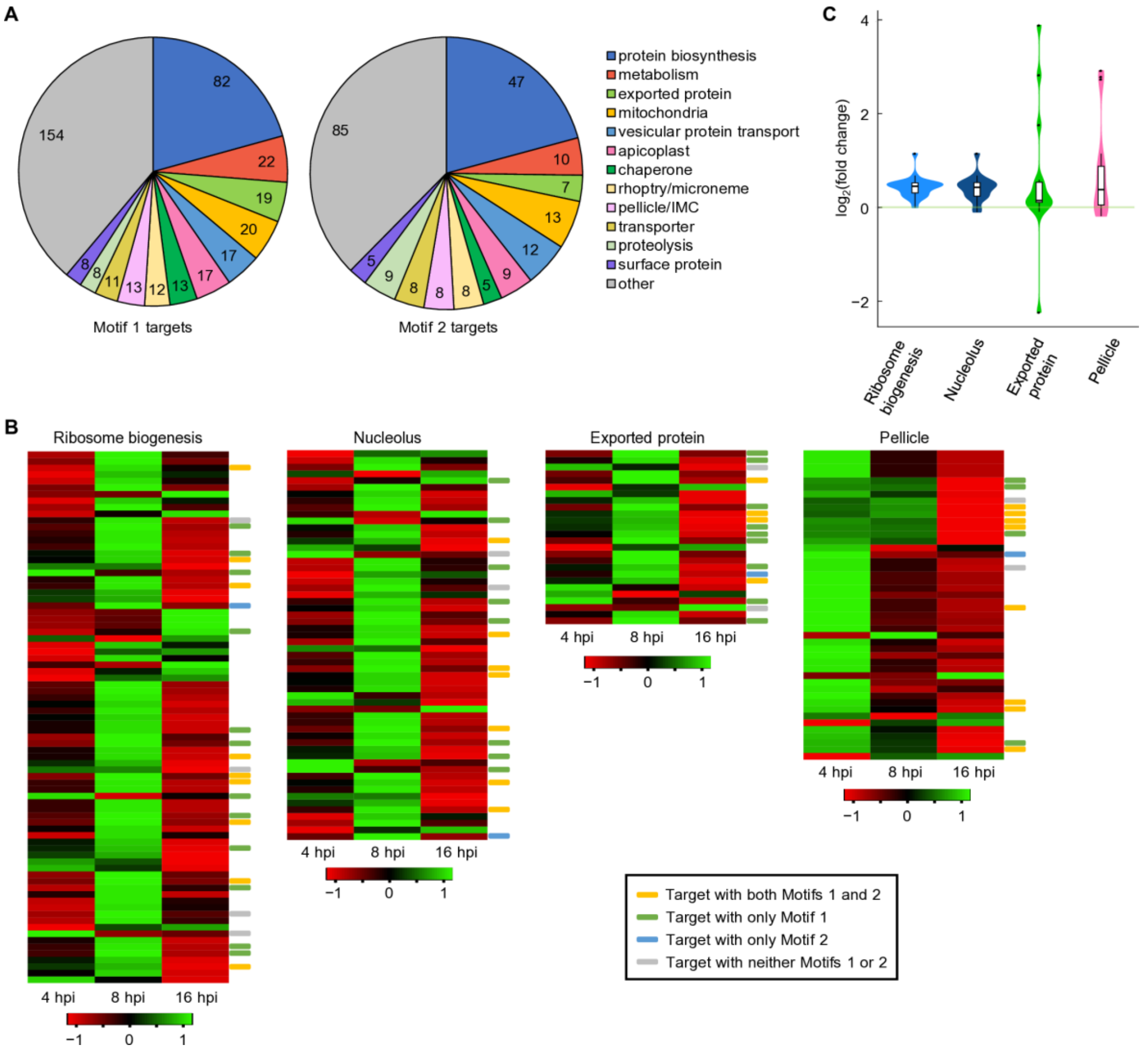

Fig. S7. Expression profile of PbAP2-TR target-enriched gene sets during the ring-to-trophozoite development.

(A) Classification of PbAP2-TR target genes that were associated with Motif 1 and 2 into 13 characteristic groups. Genes annotated on the PlasmoDB (https://plasmodb.org/plasmo/app) were classified. (B) Row-z-scored heatmaps showing expression patterns of gene sets enriched in the PbAP2-TR targets at 4 hours post-injection (hpi), 8 hpi, and 16 hpi. Target genes of PbAP2-TR with both Motifs 1and 2, only Motif 1, only Motif 2, and none of these motifs are indicated by yellow, green, blue, and grey bars, respectively, on the right side of each heatmap.

(C) Violin plot showing the distribution of log_2(fold change) values for PbAP2-TR targets in the differential expression analysis between *pbap2-tr*-DiCre^{Rapa-} and *pbap2-tr*-DiCre^{Rapa+}. Those in the target-enriched groups are shown. The horizontal line indicates log_2(fold change) of 0. The corresponding box plots show the median (center line), 25th/75th percentiles (box), and minimum/maximum values (whiskers). The dots indicate outliers.

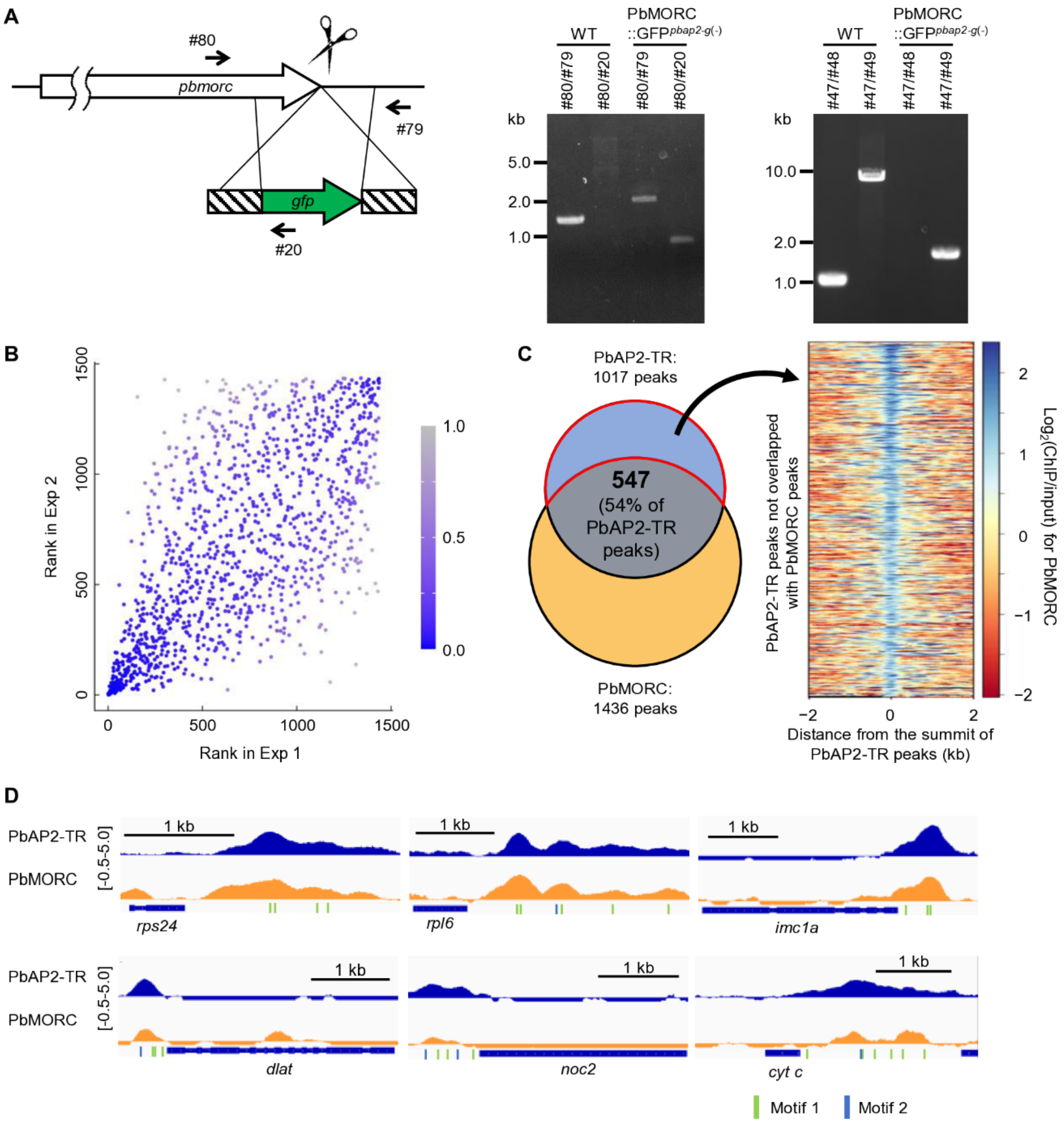

Fig. S8. Co-localization of PbMORC with PbAP2-TR on the genome.

(A) Genotyping of PbMORC::GFP$^{pbap2\text{-}g(-)}$. Schematic illustration of gene editing at the *pbmorc* locus is shown on left. Gel images from the genotyping PCR analysis for *pbmorc* and *pbap2-g* loci are shown on right. *pbap2-g* was disrupted by the same strategy illustrated in Fig S3C. The primer numbers are listed in Table S10. (B) IDR1D analysis comparing the PbMORC ChIP-seq experiments 1 and 2. The rank of peaks according to their *q*-value for each experiment is plotted against each other. The Irreproducible Discovery Rate (IDR) of each peak was represented using a gradient color scale. (C) Heatmap showing $\log_2$(ChIP/input) of PbMORC at the PbAP2-TR peaks that classified as “non-overlapping” with the PbMORC peaks under the thresholds of fold enrichment > 3 and *q*-value < 0.01 in macs2 peak calling. The heatmap was generated through the same procedure as Fig 6E, using plotHeatmap with default settings. Peak regions are aligned in the ascending order of their *q*-values. Venn diagram on left shows the number of overlapping peaks between PbAP2-TR ChIP-seq and PbMORC ChIP-seq. (D) Integrative Genomics Viewer images for the ChIP-seq experiments of PbAP2-TR and PbMORC upstream PbAP2-TR target genes (*rps24*, 40S ribosomal protein S24 gene (PBANKA_1234200); *rpl6*, 60S ribosomal protein L6 gene (PBANKA_1351900); *imc1a*, inner membrane complex protein 1a gene (PBANKA_0402600); *dlat*, dihydrolipoamide acyltransferase gene (PBANKA_0505000); *noc2*, nucleolar complex protein 2 gene (PBANKA_1353700); *cyt c*, cytochrome c gene (PBANKA_1410200)). In the PbMORC ChIP-seq, peaks upstream *rps24*, *rpl6*, and *imc1a* were detected by macs2 with fold enrichment > 3 and *q*-values < 0.01 while the other three were not with these criteria. Histograms show the $\log_2$-transformed ChIP/input ratio (bin size = 10 bp). Scales are indicated in square brackets. Positions of Motifs 1 and 2 are indicated by green and blue bars, respectively. Source data are provided as a Source Data file.
