# Supplementary material for: Precise gene regulation through transcriptional repression is essential for Plasmodium berghei asexual blood stage development
Source: Nat Commun. 2026 Jan 3;17:1508. doi: 10.1038/s41467-025-68222-1 (PMC12891473; doi:10.1038/s41467-025-68222-1)
Supplement: Supplementary file 2 — Description Of Additional Supplementary File [file 41467_2025_68222_MOESM2_ESM.pdf]

Description of Additional Supplementary Files:

Supplementary Data 1. ChIP-seq analysis of PbAP2-TR

(A) Peaks in experiment 1. (B) Peaks in experiment 2. (C) Quality check. (D) Motif enrichment analysis. *p*-values were calculated using Fisher's exact test.

Supplementary Data 2. DIP-seq analysis of PbAP2-TR

(A) DIP-seq peaks for AP2 domain 1–2. (B) Motif enrichment analysis for DIP-seq of AP2 domain 1–2. *p*-values were calculated using Fisher's exact test. (C) DIP-seq peaks for AP2 domain 3. (D) Motif enrichment analysis for DIP-seq of AP2 domain 3. *p*-values were calculated using Fisher's exact test.

Supplementary Data 3. Target genes of PbAP2-TR

Supplementary Data 4. Differential expression analysis between 8 and 16 hpi using *ap2-g*-knockout parasite

p-values adjusted for multiple testing with the Benjamini-Hochberg procedure were calculated using DESeq2.

Supplementary Data 5. Differential expression analysis between *pbap2-tr*-DiCre$^{\text{Rapa-}}$ and *pbap2-tr*-DiCre$^{\text{Rapa+}}$

p-values adjusted for multiple testing with the Benjamini-Hochberg procedure were calculated using DESeq2.

Supplementary Data 6. Gene ontology analysis of PbAP2-TR targets

p-values were calculated using Fisher's exact test on GOstats.

Supplementary Data 7 Time-course transcriptomic analysis using *ap2-g*-knockout parasite

Supplementary Data 8. RIME analysis

(A) Comparison between PbAP2-TR::GFP$^{pbap2\text{-}g(-)}$ and WT. *p*-values were calculated using a two-tailed Student’s t-test. (B) Comparison between PbMORC::GFP$^{pbap2\text{-}g(-)}$ and WT. *p*-values were calculated using a two-tailed Student’s t-test.

Supplementary Data 9. ChIP-seq analysis of PbMORC

(A) Peaks in experiment 1. (B) Peaks in experiment 2. (C) Quality check. (D) Motif enrichment analysis with all ChIP-seq peaks. *p*-values were calculated using Fisher’s exact test. (E) Motif enrichment analysis excluding peaks common with PbAP2-TR peaks. *p*-values were calculated using Fisher’s exact test.

Supplementary Data 10. List of primers used in this study
