# Supplementary material for: Precise gene regulation through transcriptional repression is essential for Plasmodium berghei asexual blood stage development
Source: Nat Commun. 2026 Jan 3;17:1508. doi: 10.1038/s41467-025-68222-1 (PMC12891473; doi:10.1038/s41467-025-68222-1)
Supplement: Supplementary file 13 — Reporting summary [file 41467_2025_68222_MOESM13_ESM.pdf]

nature portfolio

Corresponding author(s): Masao Yuda

Last updated by author(s): Nov 5, 2025

Reporting Summary

Nature Portfolio wishes to improve the reproducibility of the work that we publish. This form provides structure for consistency and transparency in reporting. For further information on Nature Portfolio policies, see our Editorial Policies and the Editorial Policy Checklist.

Statistics

For all statistical analyses, confirm that the following items are present in the figure legend, table legend, main text, or Methods section.

n/a	Confirmed	
☐	☒	The exact sample size (*n*) for each experimental group/condition, given as a discrete number and unit of measurement
☐	☒	A statement on whether measurements were taken from distinct samples or whether the same sample was measured repeatedly
☐	☒	The statistical test(s) used AND whether they are one- or two-sided<br>*Only common tests should be described solely by name; describe more complex techniques in the Methods section.*
☐	☒	A description of all covariates tested
☐	☒	A description of any assumptions or corrections, such as tests of normality and adjustment for multiple comparisons
☐	☒	A full description of the statistical parameters including central tendency (e.g. means) or other basic estimates (e.g. regression coefficient) AND variation (e.g. standard deviation) or associated estimates of uncertainty (e.g. confidence intervals)
☐	☒	For null hypothesis testing, the test statistic (e.g. *F*, *t*, *r*) with confidence intervals, effect sizes, degrees of freedom and *P* value noted<br>*Give P values as exact values whenever suitable.*
☒	☐	For Bayesian analysis, information on the choice of priors and Markov chain Monte Carlo settings
☒	☐	For hierarchical and complex designs, identification of the appropriate level for tests and full reporting of outcomes
☐	☒	Estimates of effect sizes (e.g. Cohen's *d*, Pearson's *r*), indicating how they were calculated

Our web collection on statistics for biologists contains articles on many of the points above.

Software and code

Policy information about availability of computer code

Data collection	No software was used to collect data in this study.
Data analysis	bowtie2-2.3.5.1, samtools-1.6, bedtools-2.30.0, macs2-2.1.4, hisat2-2.1.0, subread-2.0.1, deeptools-3.3.0, r-4.3.1 (DESeq2, ggplot2), GSEABase-1.64.0, GOstats-2.68.0

For manuscripts utilizing custom algorithms or software that are central to the research but not yet described in published literature, software must be made available to editors and reviewers. We strongly encourage code deposition in a community repository (e.g. GitHub). See the Nature Portfolio guidelines for submitting code & software for further information.

Data

Policy information about availability of data

All manuscripts must include a data availability statement. This statement should provide the following information, where applicable:

- Accession codes, unique identifiers, or web links for publicly available datasets
- A description of any restrictions on data availability
- For clinical datasets or third party data, please ensure that the statement adheres to our policy

All ChIP-sequencing, RNA-sequencing and DIP-sequencing data generated in this study have been deposited in the Gene Expression Omnibus database under accession numbers GSE290541 [https://www.ncbi.nlm.nih.gov/geo/query/acc.cgi?acc=GSE290541], GSE290542 [https://www.ncbi.nlm.nih.gov/geo/query/acc.cgi?acc=GSE290542], GSE290544 [https://www.ncbi.nlm.nih.gov/geo/query/acc.cgi?acc=GSE290544], and GSE290545 [https://www.ncbi.nlm.nih.gov/geo/query/

acc.cgi?acc=GSE290545]. All MS/MS data for RIME have been deposited in the ProteomeXchange Consortium under dataset PXD067426 [https://proteomecentral.proteomexchange.org/cgi/GetDataset?ID=PXD067426]. Source data are provided with this paper.

Research involving human participants, their data, or biological material

Policy information about studies with human participants or human data. See also policy information about sex, gender (identity/presentation), and sexual orientation and race, ethnicity and racism.

Reporting on sex and gender	*Use the terms sex (biological attribute) and gender (shaped by social and cultural circumstances) carefully in order to avoid confusing both terms. Indicate if findings apply to only one sex or gender; describe whether sex and gender were considered in study design; whether sex and/or gender was determined based on self-reporting or assigned and methods used. Provide in the source data disaggregated sex and gender data, where this information has been collected, and if consent has been obtained for sharing of individual-level data; provide overall numbers in this Reporting Summary. Please state if this information has not been collected. Report sex- and gender-based analyses where performed, justify reasons for lack of sex- and gender-based analysis.*
Reporting on race, ethnicity, or other socially relevant groupings	*Please specify the socially constructed or socially relevant categorization variable(s) used in your manuscript and explain why they were used. Please note that such variables should not be used as proxies for other socially constructed/relevant variables (for example, race or ethnicity should not be used as a proxy for socioeconomic status). Provide clear definitions of the relevant terms used, how they were provided (by the participants/respondents, the researchers, or third parties), and the method(s) used to classify people into the different categories (e.g. self-report, census or administrative data, social media data, etc.) Please provide details about how you controlled for confounding variables in your analyses.*
Population characteristics	*Describe the covariate-relevant population characteristics of the human research participants (e.g. age, genotypic information, past and current diagnosis and treatment categories). If you filled out the behavioural & social sciences study design questions and have nothing to add here, write "See above."*
Recruitment	*Describe how participants were recruited. Outline any potential self-selection bias or other biases that may be present and how these are likely to impact results.*
Ethics oversight	*Identify the organization(s) that approved the study protocol.*

Note that full information on the approval of the study protocol must also be provided in the manuscript.

Field-specific reporting

Please select the one below that is the best fit for your research. If you are not sure, read the appropriate sections before making your selection.

☒ Life sciences ☐ Behavioural & social sciences ☐ Ecological, evolutionary & environmental sciences

For a reference copy of the document with all sections, see nature.com/documents/nr-reporting-summary-flat.pdf

Life sciences study design

All studies must disclose on these points even when the disclosure is negative.

Sample size	Sample size calculation was not performed for any experiments in this study. Sample sizes were determined based on reported experimental designs of similar studies. ChIP-seq analyses were performed in biological duplicate to determine reproducible peak regions for further motif enrichment and target analyses. Other experiments were performed in more than three replicates, which were sufficient to perform statistical analysis, such as Student's t-test.
Data exclusions	Subtelomeric multigene families (pir and fam) were also excluded because their expression could be clonally variant.
Replication	All experiments were performed in biological replicates and showed successful replication. For ChIP-seq analyses, reproducibility was verified by assessing overlap in ChIP-seq peek locations, peak rank correlation using IDR1D (setting max gap as 100), and correlation of genome-wide read coverage using multiBigwigSummary bins (setting bin size as 1 kb).
Randomization	No experiments were randomized as samples were not allocated into multiple groups.
Blinding	All experiments did not involve group allocation, and thus blinding was not relevant to this study.

Reporting for specific materials, systems and methods

We require information from authors about some types of materials, experimental systems and methods used in many studies. Here, indicate whether each material, system or method listed is relevant to your study. If you are not sure if a list item applies to your research, read the appropriate section before selecting a response.

Materials & experimental systems

n/a	Involved in the study
☐	☒ Antibodies
☒	☐ Eukaryotic cell lines
☒	☐ Palaeontology and archaeology
☐	☒ Animals and other organisms
☒	☐ Clinical data
☒	☐ Dual use research of concern
☒	☐ Plants

Methods

n/a	Involved in the study
☐	☒ ChIP-seq
☒	☐ Flow cytometry
☒	☐ MRI-based neuroimaging

Antibodies

Antibodies used: Anti-GFP antibody, Abcam, ab290, rabbit polyclonal, Lot: GR19413-1, 1:250

Validation: The anti-GFP antibody used in this study was obtained from commercial vendors. https://www.abcam.co.jp/products/primary-antibodies/gfp-antibody-ab290.html

Animals and other research organisms

Policy information about studies involving animals; ARRIVE guidelines recommended for reporting animal research, and Sex and Gender in Research

Laboratory animals: Mouse, Mus musculus, ddY (an outbred strain purchased from Japan SLC Inc.), female, 5-6-week-old, kept in a temperature-controlled room (22–24 °C) under a 16:8 h light/dark cycle with relative humidity within 40–60%.

Wild animals: This study did not involve wild animals.

Reporting on sex: This study did not involve sex of animals.

Field-collected samples: This study did not involve samples collected from the field.

Ethics oversight: All experiments were performed in accordance with the recommendations of the Guide for the Care and Use of Laboratory Animals of the National Institutes of Health to minimize animal suffering and were approved by the Animal Research Ethics Committee of Mie University (permit number 23–29).

Note that full information on the approval of the study protocol must also be provided in the manuscript.

Plants

Seed stocks: *Report on the source of all seed stocks or other plant material used. If applicable, state the seed stock centre and catalogue number. If plant specimens were collected from the field, describe the collection location, date and sampling procedures.*

Novel plant genotypes: *Describe the methods by which all novel plant genotypes were produced. This includes those generated by transgenic approaches, gene editing, chemical/radiation-based mutagenesis and hybridization. For transgenic lines, describe the transformation method, the number of independent lines analyzed and the generation upon which experiments were performed. For gene-edited lines, describe the editor used, the endogenous sequence targeted for editing, the targeting guide RNA sequence (if applicable) and how the editor was applied.*

Authentication: *Describe any authentication procedures for each seed stock used or novel genotype generated. Describe any experiments used to assess the effect of a mutation and, where applicable, how potential secondary effects (e.g. second site T-DNA insertions, mosiacism, off-target gene editing) were examined.*

ChIP-seq

Data deposition

☒ Confirm that both raw and final processed data have been deposited in a public database such as GEO.

☒ Confirm that you have deposited or provided access to graph files (e.g. BED files) for the called peaks.

Data access links
May remain private before publication.: https://www.ncbi.nlm.nih.gov/geo/query/acc.cgi?acc=GSE290542

Files in database submission: fastq.gz files and macs2 peak files

Genome browser session
(e.g. UCSC)

https://igv.org/app/?
sessionURL=blob:zZVPb5swGMa_iw87pYlNTIBI1dR10jRt66JuO1VVZMCAwcZg889E.e7zqnRNO9r00MMuHPy.fnh.72PMDnRUa
SZLsAbLOZ4jMAM6k_0PIipOr4igGqwTwjWdAUUTqmgZUbDegYTohvy6_mr3ZU1T6fVi0ff9PFayCuUwj6RY6IgvErbghpkUtooQyj
BTDEtN0WLDiRby44czvNpuQqrSjLKLqy8X20.0lILO7_TfK15Qc140iUBRm_Qh930nM0s_hsUo2_BdzM.hdczKmA6vMiNIp5O88B
qUCu2IxudhQl5nxj7ZwVBDhb_kZZyOJa9NHPi07FeOGsa_huL_YC77GeAyam2AgHBuXSnJwPrmdgYaRaLCrt_sQGMqmyfQtG7vo
p0BqWKqwPosgNBDQeC42MMwCNB.tgOt4qfByqx3Iaq1DHOf1YOqcdTCxSa82DhnP6.3l9nnzRbNQxqnilTZgUVhUuGBGpHnKeF
0zDQ0BEcG3o80YZyW9jza9z8nZbtOdxzwsFWUSpDGdh9VD.PoWQpezRt3I2WUkdR3q0FkuYowLJ_wOk958xQFqcfGVQzrFQ.KS
mWtqRDvT_I6J3mdCV737XghVMRxNCSchI5JdFc6xcryfvt.fflMukOHuhx3Y03yoIi5qfoxWFYSZpO0U0LHrM_UD6SrtyNFBaKuRjm
OMAmxX6OKmuYR6T.5VlwsXTF0JmztCS4hzh2Jo05P5zolNE06lan3dqReJzIWhDWK6zYwbq3cZAgfLiPXfXQZzdMkuf9ulYg839Q4
HutoTCNSR3ngyxBP8b4gd0T9cteB3T9it4XlAzcpS9mQ5s8_bQbs3jSzPR7c3.5_Aw--

Methodology

Replicates

Biological duplicate

Sequencing depth

For all experiments, samples were sequenced in 150-bp paired-end mode. Number of uniquely and concordantly mapped reads/total number of reads for each experiments is as follows: PbAP2-TR_ChIP1, 4,901,117/12,339,159 reads; PbAP2-TR_input1, 8,522,677/11,944,977 reads; PbAP2-TR_ChIP2, 5,298,340/13,227,795 reads; PbAP2-TR_input2, 8,315,765/12,286,177 reads; PbMORC_ChIP1, 7,283,084/12,041,264 reads; PbMORC_input1, 4,669,587/6,954,551 reads; PbMORC_ChIP2, 12838560 reads; PbMORC_input2, reads.

Antibodies

Anti-GFP antibody, Abcam, ab290, polyclonal, GR19413-1

Peak calling parameters

Reads were mapped on the P. berghei genome using Bowtie 2.0 with default settings. The reads mapped onto multiple sites were removed using grep command. The SAM files were sorted using samtools to produce BAM files. The mapping data were analyzed with the MACS2 peak-calling algorithm with the --call-summits option. Peaks were called with fold enrichment > 3.0, q-value < 0.01 using sequence data of input DNA as a control.

Data quality

To assess data qualities, fraction of reads in peaks (FRiP), normalized strand cross-correlation coefficient (NSC), and relative strand cross-correlation coefficient (RSC) were calculated using plotEnrichment (deepTools) and phantompeakqualtools. Reproducibility was verified using IDR1D (https://idr2d.mit.edu/) and multiBigwigSummary bins (deepTools). The IDR1D analysis showed consistency of peak ranks between duplicate with most peaks having idr < 0.1. The genome-wide read coverage comparison resulted in Pearson correlation coefficient of 0.96 and 0.94 for PbAP2-TR and PbMORC ChIP-seq, respectively.

Software

bowtie2-2.3.5.1, samtools-1.6, bedtools-2.30.0, macs2-2.1.4, deeptools-3.3.0.
